# Combined Antagonism of 5-HT_2_ and NMDA Receptors Reduces the Aggression of Monoamine Oxidase a Knockout Mice

**DOI:** 10.3390/ph15020213

**Published:** 2022-02-10

**Authors:** Roberto Frau, Alessandra Pardu, Sean Godar, Valentina Bini, Marco Bortolato

**Affiliations:** 1 Department of Biomedical Sciences, Division of Neuroscience and Clinical Pharmacology, University of Cagliari, 09042 Monserrato, Italy; apardu@gmail.com (A.P.); vbini2022@gmail.com (V.B.); 2 Guy Everett Laboratory, Department of Biomedical Sciences, Division of Neuroscience and Clinical Pharmacology, University of Cagliari, 09042 Monserrato, Italy; 3 Department of Pharmacology and Pharmaceutical Sciences, University of Southern California, Los Angeles, CA 90089, USA; scgodar22@gmail.com; 4 Department of Pharmacology and Toxicology, College of Pharmacy, University of Utah, Salt Lake City, UT 84112, USA

**Keywords:** monoamine oxidase A, NMDA receptors, 5-HT_2_ receptors, aggression, prepulse inhibition

## Abstract

The enzyme monoamine oxidase A (MAOA) catalyzes the degradation of several neurotransmitters, including serotonin. A large body of evidence has shown that genetic MAOA deficiency predisposes humans and mice to aggression and antisocial behavior. We previously documented that the aggression of male MAOA-deficient mice is contributed by serotonin 5-HT_2_ and glutamate N-methyl-D-aspartate (NMDA) receptors in the prefrontal cortex (PFC). Indeed, blocking either receptor reduces the aggression of MAOA knockout (KO) mice; however, 5-HT_2_ receptor antagonists, such as ketanserin (KET), reduce locomotor activity, while NMDA receptor blockers are typically associated with psychotomimetic properties. To verify whether NMDA receptor blockers induce psychotomimetic effects in MAOA KO mice, here we tested the effects of these compounds on prepulse inhibition (PPI) of the acoustic startle reflex. We found that male MAOA KO mice are hypersensitive to the PPI-disrupting properties of NMDA receptor antagonists, including the non-competitive antagonist dizocilpine (DIZ; 0.1, 0.3 mg/kg, IP) and the NR2B subunit-specific blocker Ro-256981 (5, 10 mg/kg, IP). Since KET has been previously shown to counter the PPI deficits caused by NMDA receptor antagonists, we tested the behavioral effects of the combination of KET (2 mg/kg, IP) and these drugs. Our results show that the combination of KET and DIZ potently reduces aggression in MAOA KO mice without any PPI deficits and sedative effects. While the PPI-ameliorative properties of KET were also observed after infusion in the medial PFC (0.05 μg/side), KET did not counter the PPI-disruptive effects of Ro-256981 in MAOA KO mice. Taken together, these results point to the combination of non-subunit-selective NMDA and 5-HT_2_ receptor antagonists as a potential therapeutic approach for aggression and antisocial behavior with a better safety and tolerability profile than each monotherapy.

## 1. Introduction

Antisocial behavior is characterized by aggressive and non-aggressive rule-breaking, violation of societal norms, disregard for the rights and properties of others, and violence [1]. Although the robust association between pathological aggression, antisocial behavior, and delinquency imposes a hefty burden on society [2], preventive and therapeutic interventions for these problems remain highly challenging. To date, no drug has been approved for its treatment [3,4]. Several studies have investigated the biological underpinnings of pathological aggression and antisocial behavior. To date, the best-characterized molecular factor associated with these disorders is monoamine oxidase A (MAOA), the key enzyme catalyzing the metabolism of serotonin and norepinephrine [5,6,7]. In men, *MAOA* genetic deficiency leads to Brunner syndrome, a condition characterized by overt aggression, antisocial conduct, and autism-related intellectual disabilities [8,9]. Likewise, male MAOA knockout (KO) mice exhibit a spectrum of behavioral alterations strikingly congruent with those observed in Brunner syndrome, including high aggression, socio-communicative deficits, and maladaptive and perseverative responses [10,11,12].

We previously showed that the core phenotypic features of MAOA KO mice are accompanied by alterations of N-methyl-d-aspartate (NMDA) glutamate receptors in the forebrain [13]. These receptors serve a crucial function in regulating information processing [14] and aggression [15]. NMDA receptors are ionotropic channels formed by different subunits, including NR1 and the four members of the NR2 family [16]. We documented that the medial prefrontal cortex (mPFC) of MAOA KO mice displays a significant increase in the expression of NR2A and, to an even greater extent, NR2B subunits [13]. The same changes have been recently identified in neurons derived from pluripotent stem cells from Brunner syndrome patients [17]. In MAOA KO mice, we showed that these alterations biased the biophysical properties of NMDA receptors, resulting in a marked reduction in decay time and excitability [13]. Most importantly, low doses of NMDA receptor blockers, such as dizocilpine (DIZ) or the NR2B-specific blocker Ro-256981, curbed the aggression exhibited by MAOA KO mice [13]; these effects were specific, as they were not shared by their wild-type (WT) littermates. These results may have profound implications: on one hand, they point to NMDA receptor deficits as a critical mechanism underlying the aggression exhibited by MAOA-deficient individuals; on the other hand, they point to NMDA receptor blockers as selective, highly effective anti-aggressive therapies.

Overall, this research points to NMDA receptors as key mediators of the behavioral outcomes of MAOA deficiency. Most importantly, while the effects of NMDA receptor antagonists are promising, the therapeutic potential of these compounds is greatly limited by their psychotomimetic properties [18]. In animal models, the psychotomimetic effect of dizocilpine and other NMDA receptor antagonists is best studied by deficits of the prepulse inhibition (PPI) of the startle reflex. For example, DIZ and Ro-256981 have been shown to produce major PPI impairments in rodents [19,20]. Notably, one of the most efficacious tools to counter the PPI deficits produced by NMDA receptor blockers is the antagonism of 5-HT_2_ receptors, such as ketanserin (KET) [21]. The ability of mPFC 5-HT_2_ receptors to control NMDA-mediated pyramidal excitability and influence emotional and cognitive functions likely contributes to this effect [22]. Notably, KET also reverses and prevents aggressive behavior in MAOA KO mice [23]. Nevertheless, KET also reduces the locomotor activity in both MAOA KO and WT mice, raising the caveat that the observed anti-aggressive effects may be non-specific [23].

Based on these premises, we tested whether doses of NMDA receptor blockers that reduce aggression in MAOA KO mice may also elicit PPI deficits and tested whether the combination of these drugs and KET may preserve anti-aggressive effects in MAOA KO mice without PPI impairments.

## 2. Results

### 2.1. Low Doses of NMDA Receptor Antagonists DIZ and Ro 25-6981 Impair PPI in MAOA KO, but Not WT Mice

We previously showed that low doses of DIZ and Ro 25-6981 potently reduced aggression in MAOA KO, but not WT mice. Given that these two compounds have also been associated with PPI disruption [19,20,24], we tested whether the same doses induced PPI deficits in MAOA-deficient mice.

**Figure 1:**
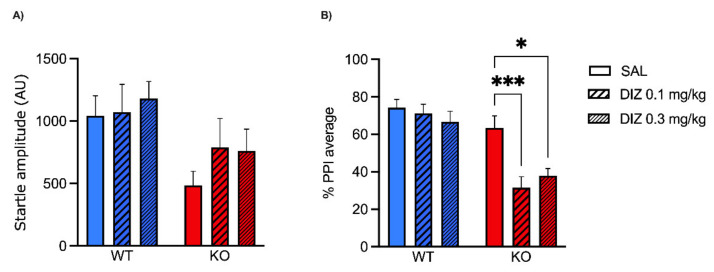
Effects of the uncompetitive NMDA receptor antagonist dizocilpine (DIZ; 0.1, 0.3 mg/kg, IP) on startle reflex (**A**) and prepulse inhibition (**B**) in MAOA KO mice. Data are shown as means ± SEM. Significance levels refer to the results of post hoc comparisons of two-way ANOVA analyses. Main effects are not indicated. * *p* < 0.05, *** *p* < 0.001 for comparisons indicated by bracket lines. (SAL, saline; AU, arbitrary units); *n* = 6–10/group. For further details, see results section.

In the first experiment, WT (*n* = 18) and MAOA KO mice (*n* = 25) were injected with either saline or DIZ (0.1, 0.3 mg/kg, IP) and, after 10 min, were subjected to startle and PPI sessions. Startle magnitudes were evaluated using a two-way ANOVA (genotype and treatment as between-subjects factors). ANOVA detected a significant main effect of genotype (F(1, 37) = 6.82, *p* < 0.05) due to the overall reduction in startle amplitude exhibited by MAOA KO mice (Figure 1A). Conversely, ANOVA did not evidence either a main effect of DIZ treatment (F(2, 37) = 0.59, *p* = 0.55, NS) nor a genotype × treatment interaction (F(2, 37) = 0.24, *p* = 0.78, NS). PPI was also evaluated by a similar two-way ANOVA design. As indicated in Figure 1B, a significant genotype × treatment interaction was found (F(2, 37) = 3.47; *p* < 0.05). Post hoc analyses documented that DIZ significantly disrupted MAOA KO PPI, but not their WT counterparts. In detail, Tukey’s post hoc test revealed that both doses of DIZ produced robust PPI deficits in MAOA KO, but not in WT counterparts (*p* < 0.001 and *p* < 0.05 for comparisons between MAOA KO treated with saline and MAO KO treated with the low and high dose of DIZ, respectively; Tukey’s test). 

**Figure 2:**
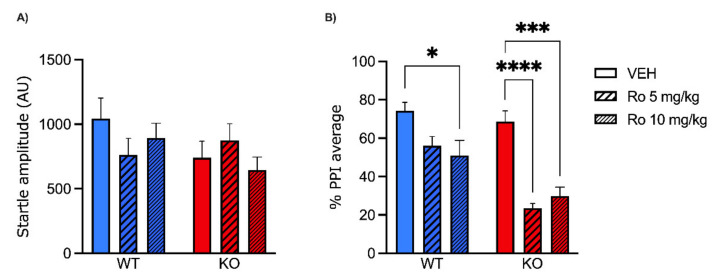
Effects of the NR2B subunit-specific antagonist Ro-256981 (5, 10 mg/kg, IP) on startle reflex (**A**) and prepulse inhibition (**B**) in MAOA KO mice. Data are shown as means ± SEM. Significance levels refer to the results of post hoc comparisons of two-way ANOVA analyses. Main effects are not indicated. * *p* < 0.05, *** *p* < 0.001, **** *p* < 0.0001 for comparisons indicated by bracket lines. (SAL, saline; AU, arbitrary units); *n* = 6/group. For further details, see results section.

In the second experiment, we used the same design to test the effect of Ro 25-6981 (5, 10 mg/kg, IP) on PPI. WT (*n* = 18) and MAOA KO (*n* = 18) mice received Ro 25-6981 (or vehicle) 10 min before startle and PPI evaluation. Startle analysis (Figure 2A) identified no significant effects of either genotype (F(1, 30) = 1.95; NS, ANOVA) or treatment (F(2, 30) = 0.46; NS, ANOVA). Furthermore, no significant interaction between these two factors was found (F(2, 30) = 1.53; *p* = 0.23, NS, ANOVA). The analysis of PPI values revealed a significant interaction between genotype and treatment (F(2, 30) = 3.41; *p* < 0.05, ANOVA). Tukey’s test revealed that while only the higher dose of Ro 25-6981 (10 mg/kg) elicited a significant PPI deficit in WT mice (*p* < 0.05), both doses reduced PPI values in MAOA KO mice (*p* < 0.0001 for 5 mg/kg and *p* < 0.001 for 10 mg/kg). These data indicate that MAOA deficiency increases the sensitivity to the PPI-disrupting effects of NR2B-selective NMDA receptor antagonism (Figure 2B).

### 2.2. KET Rescues the PPI Deficits Induced by DIZ, but Not Ro 25-6981, in MAOA KO Mice

The next experiment aimed to investigate whether the systemic administration of KET may counteract the PPI deficits induced by DIZ in MAOA KO mice. Mice (WT: *n* = 22; MAOA KO: *n* = 27) were administered either KET (2 mg/kg, IP) or vehicle 20 min before DIZ (0.1 mg/kg) injection; 10 min after DIZ administration, animals were subjected to PPI test. Analyses were run by three-way ANOVAs, with genotype (WT or MAOA KO), KET (vs. its vehicle), and DIZ (vs. saline) as independent variables.

**Figure 3:**
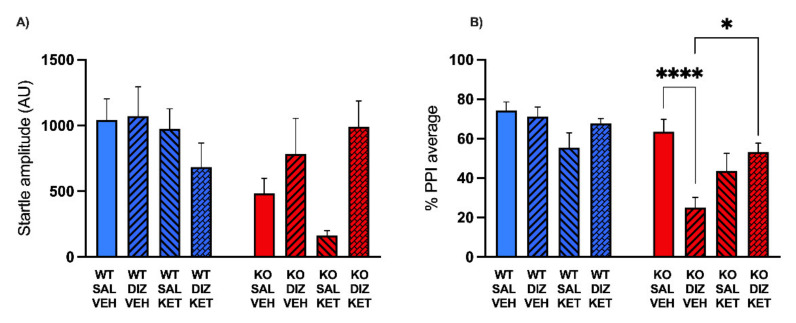
Effects of the systemic administration of ketanserin (KET, 2 mg/kg, IP) on the changes in startle reflex (**A**) and prepulse inhibition (**B**) induced by dizocilpine (DIZ, 0.1mg/kg, IP) in MAOA KO mice. Data are shown as means ± SEM. Significance levels refer to the results of post hoc comparisons of three-way ANOVA analyses. Main effects are not indicated. * *p* < 0.05, **** *p* < 0.0001 for comparisons indicated by bracket lines. (SAL, saline; VEH, vehicle; AU, arbitrary units); *n* = 6–8/group. For further details, see results section.

The analysis of startle amplitude revealed a significant main effect of genotype (F(1, 41) = 5.14; *p* < 0.05, ANOVA) due to the reduction in startle magnitude in MAOA KO mice. However, neither KET nor DIZ led to significant changes in startle amplitude (KET: F(1, 41) = 0.92; NS; ANOVA; DIZ: F(1, 41) = 2.10; NS; ANOVA); furthermore, a significant interaction between genotype and DIZ was found (F(1, 41) = 5.46; *p* < 0.05). Post hoc analyses detected a significant difference between WT and MAOA KO treated with saline, but not between DIZ-treated mice (WT-SAL vs. WT-DIZ, *p* = 0.91, NS; KO-SAL vs. KO DIZ *p* = 0.52, NS). No other interactions across factors were significant (Figure 3A). A further three-way ANOVA tested PPI values. The analysis revealed a significant main effect of genotype (F(1, 41) = 27.29; *p* < 0.0001) relative to an overall reduction in PPI in MAOA KO mice, but not of either KET (F(1, 41) = 0,79; NS) or DIZ (F(1, 41) = 1.48; N.S.). ANOVA also disclosed a three-way interaction (F(1, 41) = 4.20; *p* < 0.05). Post hoc comparisons revealed that DIZ significantly impaired PPI in MAOA KO mice, and KET rescued this deficit (KO-VEH-SAL vs. KO-VEH-DIZ, *p* < 0.0001; KO-VEH-DIZ vs. KO-KET-DIZ, *p* < 0.01, Tukey’s) (Figure 3B).

**Figure 4:**
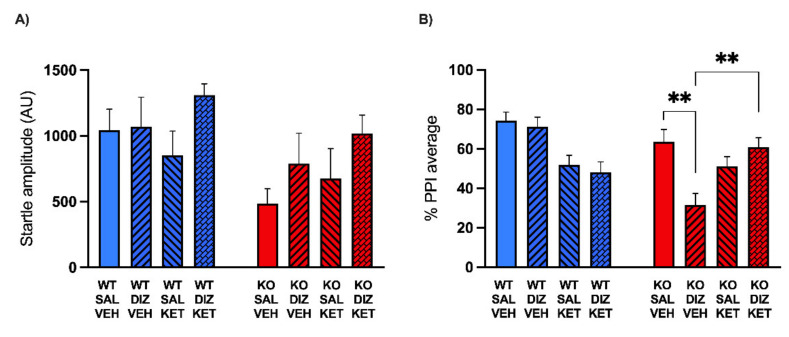
Effects of the medial prefrontal cortex (mPFC) infusion of ketanserin (KET, 0.05 μg/side) on the changes in startle reflex (**A**) and prepulse inhibition (**B**) induced by dizocilpine (DIZ, 0.1mg/kg, IP) in MAOA KO mice. Data are shown as means ± SEM. Significance levels refer to the results of post hoc comparisons of three-way ANOVA analyses. Main effects are not indicated. ** *p* < 0.01 for comparisons indicated by bracket lines. (SAL, saline; VEH, vehicle; AU, arbitrary units); *n* = 6–10/group. For further details, see results section.

Following this experiment, we studied whether the effects of systemic KET may also be observed following its injection in the mPFC (0.05 μg/side), given that this area exhibits alterations in MAOA subunit composition and biophysical properties [13]. WT (*n* = 27) and MAOA KO mice (*n* = 32) were injected with DIZ (or vehicle) 10 min before infusing KET into the mPFC. After the intra-PFC administration of KET, animals were tested for PPI session. ANOVA disclosed a significant main effect of genotype (F(1, 51) = 6.13; *p* < 0.05, ANOVA) and DIZ (F(1, 51) = 4.60; *p* < 0.05, ANOVA) on startle amplitude values, indicating a reduction and increase in startle magnitude in MAOA KO mice and after DIZ administration, respectively. Conversely, no main effect of KET (F(1, 51) = 0.79; NS, ANOVA) or interactions across factors (F(1, 51) = 0.54; NS, ANOVA) were found (Figure 4A). The analysis of PPI revealed a significant main effect of the genotype (F(1, 51) = 6.18; *p* < 0.05, ANOVA), but not of the other factors KET (F(1, 51) = 3.49; NS, ANOVA) and DIZ (F(1, 51) = 3.46; NS, ANOVA). ANOVA also detected a significant three-way interaction (F(1, 51) = 7.55; *p* < 0.01, ANOVA). Multiple comparisons indicated that the subthreshold dose of DIZ disrupted PPI in MAOA KO mice, but not in WT (KO-VEH-SAL vs. KO-VEH-DIZ, *p* < 0.01, Tukey’s test), and that the infusion of KET in the mPFC rescued PPI deficits of MAOA KO mice induced by DIZ administration (KO-VEH-DIZ vs. KO-KET-DIZ, *p* < 0.01, Tukey’s test) (Figure 4B).

The last experiment evaluated whether the intra-mPFC infusions of KET (0.05 μg/side) rescued the PPI deficits induced by Ro 25-6981 (5 mg/kg, IP), the selective blockers of NR2B-containing NMDA receptors. 

WT (*n* = 27) and MAOA KO mice (*n* = 32) were injected with Ro 25-6981 (or vehicle) 10 min before receiving the local infusion of KET into the mPFC. After KET administration, mice were subjected to PPI session. 

**Figure 5:**
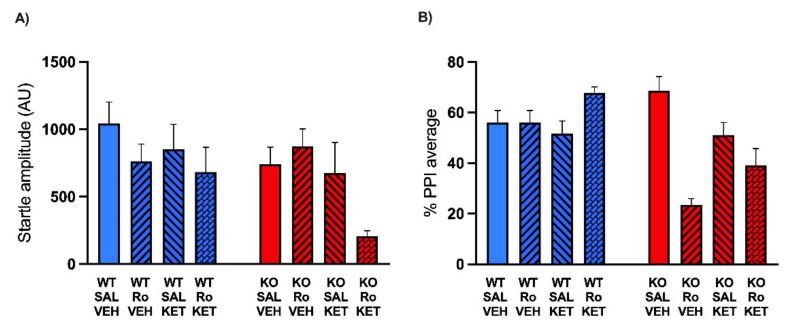
Effects of the medial prefrontal cortex (mPFC) infusion of ketanserin (KET, 0.05 μg/side) on the changes in startle reflex (**A**) and prepulse inhibition (**B**) induced by Ro-256981 (5 mg/kg, IP) in MAOA KO mice. Data are shown as means ± SEM. Significance levels refer to the results of post hoc comparisons of three-way ANOVA analyses. Main effects are not indicated. (SAL, saline; VEH, vehicle; AU, arbitrary units); *n* = 6–7/group. For further details, see results section.

Startle amplitudes were evaluated with a three-way ANOVA design (Figure 5A). ANOVA showed that KET reduced startle magnitudes (Main KET effect: F(1, 42) = 4.75; *p* < 0.05, ANOVA). Conversely, no main effect was detected for genotype (F(1, 42) = 3.35; *p* = 0.07, NS) or Ro 25-6981 (F(1, 42) = 2.89; *p* = 0.09, NS). Finally, ANOVA detected no significant interactions across factors. PPI values were also analyzed by three-way ANOVA (Figure 5B). Significant main effects were found for genotype (F(1, 42) = 24.94; *p* < 0.0001) and Ro 25-6981 (F(1, 42) = 19.30; *p* < 0.0001, ANOVA), but not for KET (F(1, 42) = 0.88; *p* = 0.35, NS). While ANOVA detected no significant three-way interaction (F(1, 42) = 0.002; *p* = 0.96, NS), two-way interactions between genotype X Ro 25-6981 (F(1, 42) = 16.58; *p* < 0.001) and KET X Ro 25-6981 (F(1, 42) = 24.91; *p* < 0.0001) were found. Multiple comparisons disclosed that DIZ elicited PPI deficits in MAOA KO mice, but not in their counterpart (KO-SAL vs. KO-DIZ, *p* < 0.001; WT-SAL vs. WT-DIZ, *p* = 0.99, Tukey’s) and that KET failed to reverse Ro 25-6981-mediated PPI deficits (VEH-SAL vs. VEH-DIZ, *p* < 0.0001; VEH-DIZ vs. DIZ-KET, *p* = 0.17, NS). 

### 2.3. The Combination of KET and DIZ Reduces the Aggression of MAOA KO Mice without Significant Changes of Locomotor Activity

Based on these results, we tested the combined effects of KET and DIZ on the aggression of MAOA KO mice (*n* = 48), as compared with WT littermates (*n* = 47). 

**Figure 6:**
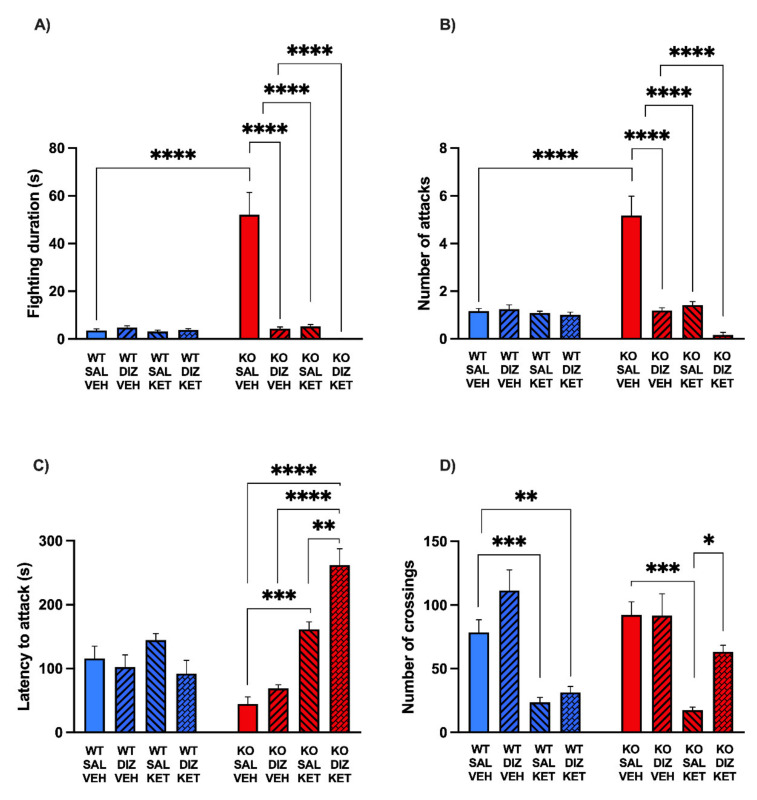
Impact of the systemic injection of ketanserin (KET, 2 mg/kg, IP) on the anti-aggressive effects of dizocilpine in MAOA KO mice. Data are shown as means ± SEM. Significance levels refer to the results of post hoc comparisons of three-way ANOVA analyses. (**A**): Overall duration of aggressive behavior; (**B**): Number of attacks; (**C**): The combination of DIZ and KET in MAOA KO mice had a significantly greater effect on latency than either drug; (**D**): motor activity within the same experiment revealed a significant three-way interaction. **** *p* < 0.0001, *** *p* < 0.001, ** *p* < 0.01, * *p* < 0.05 for comparisons indicated by bracket lines. (SAL, saline; VEH, vehicle; AU, arbitrary units); *n* = 11–12/group. Main effects are not indicated. For further details, see results section.

The analysis of the overall duration of aggressive behavior (Figure 6A) was performed by a three-way ANOVA, which detected a significant genotype × KET × DIZ interaction (F(1, 87) = 20.16, *p* < 0.0001). Post hoc comparisons revealed that MAOA KO mice treated with the vehicle of KET and saline displayed significantly longer aggression than their WT counterparts (*p* < 0.0001); furthermore, DIZ, KET, and their combination significantly reduced the duration of aggression (Ps < 0.0001 for all comparisons vs. KO-VEH-SAL). These results were confirmed by the analysis of the number of attacks (Figure 6B); indeed, ANOVA pointed to a three-way interaction (F(1, 87) = 10.58, *p* < 0.01), confirming that, while MAOA KO mice exhibited a spontaneous increase in fighting encounters (*p* < 0.0001 for the comparison between WT and KO treated with VEH and SAL), this augmentation was potently countered by both KET, DIZ, and their combination (Ps < 0.0001 for all comparisons). Post hoc comparisons further confirmed significant differences between MAOA KO mice treated with saline and vehicle and all other groups (Ps < 0.0001). A significant genotype × KET × DIZ interaction was also detected for the latency to the first attack (F(1, 87) = 5.80, *p* < 0.05; ANOVA). Post hoc comparisons revealed that WT mice had a marginally longer latency to the first attack than KO counterparts (*p* = 0.06). Neither DIZ nor KET affected the latency to aggression in WT mice; conversely, both KET and its combination with DIZ significantly increased this parameter in MAOA KO mice (KO-VEH-SAL vs. KO-KET-SAL, *p* < 0.001; KO-VEH-SAL vs. KO-KET-DIZ, *p* < 0.0001). Interestingly, the combination of DIZ and KET in MAOA KO mice had a significantly greater effect on latency than either drug (KO-VEH-DIZ vs. KO-KET-DIZ, *p* < 0.0001; KO-KET-SAL vs. KO-KET-DIZ, *p* < 0.01, Tukey’s test) (Figure 6C). The analysis of motor activity within the same experiment revealed a significant three-way interaction (F(1, 87) = 7.04, *p* < 0.01) (Figure 6D). Post hoc comparisons revealed no difference in locomotor activity between WT and KO mice (*p* = 0.98). KET reduced locomotor activity in both WT (*p* < 0.01) and KO mice (*p* < 0.001). Interestingly, while DIZ did not significantly affect the locomotor activity in either genotype, it reversed the hypolocomotion induced by KET in KO mice (*p* < 0.05), but not in WT counterparts. Accordingly, the combination of KET and DIZ reduced the locomotor activity in WT (*p* < 0.01) but not in KO mice (*p* = 0.45, NS). Overall, these data indicate that the combination of KET and DIZ, while effective in reducing aggression in KO mice, did not lessen either motor activity or PPI, like KET and DIZ alone, respectively.

## 3. Discussion

The results of this study showed that MAOA KO mice are highly susceptible to the detrimental effects of NMDA receptor antagonism on PPI. Indeed, the systemic administration of low doses of the potent non-competitive NMDA receptor antagonists DIZ elicited robust PPI deficits in MAOA KO mice but not in their WT counterparts. These results are in striking alignment with our previous finding that MAOA KO mice exhibit functional alterations of NMDA receptors (including a marked increase in NR2A and NR2B subunits and a reduction in conductance) [13]. Notably, the same modifications in NR2B subunits were identified in a recent study on neurons from individuals affected by Brunner syndrome, the clinical condition characterized by MAOA nonsense deficiency [17].

The effects of DIZ were blocked by both the systemic and intra-mPFC administration of the 5-HT_2_ receptor antagonist, KET, which rescued the PPI loss induced by DIZ. Most importantly, the combination of KET and DIZ fully ablated the high levels of aggressive behavior in MAOA KO mice but, unlike KET alone, did not significantly modify locomotor activity. These results collectively extend previous evidence from our group and others, indicating that NMDA and 5-HT_2_ receptors in the PFC are critically important in the abnormal aggressive behavior of MAOA-deficient mice [13,25]. Nevertheless, while NMDA and 5-HT_2_ receptor blockers elicit concurring anti-aggressive effects, these drugs reciprocally cancel some of the adverse behavioral effects associated with their use, namely deficits in PPI and locomotor activity. 

Taken together, our results are in striking alignment with previous evidence indicating that alterations of glutamate neurotransmission mediated by NMDA receptors in the PFC play a critical role in the expression of maladaptive behavioral responses, including pathological aggression, behavioral flexibility, and cognitive deficits [23,26,27,28]. In the PFC, NMDA receptors are abundantly expressed both on GABAergic interneurons and glutamatergic pyramidal cells. They ensure optimal excitation and inhibition (E/I) balance and maintain the efficiency of cortical information processing [29,30]. NMDA receptors are instrumental for activating PFC pyramidal neurons, allowing integration of sensory and motor information from subcortical regions to gate salient stimuli from irrelevant ones [31]. The NMDA receptor serves a crucial function in regulating information processing between PFC and subcortical structures involved in cognitive and affective domains [14,32,33,34]. The PFC-specific disruption of the NMDA receptor is accompanied by PPI deficits [35,36,37,38]. 

PPI is an operational index of sensorimotor gating, the information-processing domain aimed at filtering out irrelevant or redundant stimuli [39]. Notably, the PPI deficits induced by NMDA receptor blockers are generally interpreted to be an operational parameter to measure their psychotomimetic effects, given that these impairments are strikingly akin to the PPI deficits displayed by schizophrenia patients [40] and they are countered by antipsychotic drugs [41]. Indeed, 5-HT_2A_ receptor blockade is regarded as one of the main molecular mechanisms underlying the effects of atypical antipsychotics [42,43]; accordingly, selective 5-HT_2A_ receptor antagonists exert antipsychotic effects [44] and counter the PPI deficits induced by NMDA receptor blockers and other psychotomimetic substances [45,46,47]. Given that MAOA-deficient mice display high serotonin levels in the forebrain [11,23], our results suggest that these animals also exhibit a tonic hyperactivation of 5-HT_2A_ receptors in the mPFC, which contributes to both their aggression as well as their hypersensitivity to the psychotomimetic effects of NMDA receptor blockers. However, our results also indicate that 5-HT_2A_ receptor antagonism cancels the psychotomimetic but not the anti-aggressive effects of DIZ, suggesting that distinct neurobiological mechanisms mediate these behavioral properties. Notably, 5-HT_2A_ receptors may modulate NMDA receptor signaling through a different mechanism mediated by multiple proteins of the postsynaptic density (PSD), the core component of the network of NMDA receptors. For example, the protein PSD95, which is essential to stabilize the surface and synaptic expression of NMDA receptors [48,49], directly interacts with 5-HT_2A_ receptors and contributes to its role in behavioral regulation, including the ontogeny of hallucinations [50,51]. Depending on the specific reciprocal interaction between 5-HT_2A_ and NMDA receptors, their actions may either converge or diverge with respect to different behavioral properties. Future studies will be necessary to evaluate whether PSD 95 or other signaling proteins are implicated in the specific regulation of the crosstalk between 5-HT_2A_ and NMDA receptors. From this perspective, it is interesting to note that intra-mPFC KET administration fully countered the PPI impairments induced by DIZ, but not by the NR2B-selective NMDA receptor antagonist Ro-256981. A possible explanation for this divergence is that 5-HT_2A_ receptors may control the effects of different subtypes of NMDA receptors. From this perspective, it is worth noting that the PPI deficits induced by DIZ are reversed by the activation of NMDA receptors containing NR2C and NR2D subunits [52]. 

The deficiency of MAOA expression results in homeostatic imbalances of serotonin, along with a broad set of behavioral aberrations both in humans and rodents [6,7,53]. In humans, deficits in brain MAOA levels lead to a higher predisposition to aggressiveness and antisocial personality, as well as perseverative behavioral patterns and mild cognitive deficits [7,54,55]. Likewise, MAOA KO mice exhibit abnormal phenotypes, including marked reactive aggression towards intruder conspecifics, maladaptive reactivity to environmental cues, and autism-related phenotypes [12,56,57]. Despite this well-established relationship, the underlying neural bases for the spectrum of behavioral abnormalities associated with MAOA disruption remain unknown. The serotoninergic effects of KET in MAOA KO mice agree with prior evidence from our group and others showing that this drug and other 5-HT_2A_ receptor antagonists reverse the behavioral abnormalities of both MAOA KO and hypomorphic mice [23,25,58]. It is worth noting that we recently showed that 5-HT_2A_ receptor activation also mediates the best-known gene × environment interaction in pathological aggression and antisocial behavior, involving low-activity MAOA genotype and early postnatal stress [25]. While these data point to 5-HT_2A_ receptor blockade as a highly effective strategy to prevent the ontogeny and reduce the intensity of pathological aggression, the use of KET is generally limited by its adverse effects, including hypotension, tiredness, and dizziness, which are related to its sedative and hypotensive properties [59]. DIZ may have counteracted these effects, given that low doses of this and other NMDA receptor blockers have often been associated with hyperactivity [60,61,62]. From this perspective, our results suggest that, while the antagonism of NMDA and 5-HT_2A_ receptors are not ideal strategies to reduce pathological aggression, their combination may be a valuable approach to counter this behavioral disturbance. 

Several limitations of this study must be acknowledged. First, this study tested only MAOA KO mice and their WT controls. While MAOA KO mice exhibit alterations akin to those displayed by individuals with Brunner syndrome, this genetic condition is rare; thus, the present findings cannot yet be fully generalized to other forms of aggression associated with low-activity MAOA genetic variants or reduced enzyme activity. That said, previous studies by our group have begun validating the idea that 5-HT_2A_ receptors may be implicated in other forms of aggression driven by hypomorphic *MAOA* genotypes [25]. Furthermore, the binding of this receptor was found to be increased in the prefrontal cortex of aggressive individuals (irrespective of their genotype) [63], raising the possibility that at least some of the present findings may be generalized to multiple forms of pathological aggression. Future studies are warranted to test the applicability of this combined pharmacological approach to other mouse models of aggression before any conclusion can be drawn about the viability of this strategy for the reduction of aggressive manifestations in humans. Second, we only assessed behavioral outcomes in male mice. While the literature indicates that female mice also tend to engage in some forms of aggressive behavior, the association between *MAOA* genotypes and aggression in females is not yet fully corroborated by the available evidence [7]. That said, future investigations are needed to establish whether the behavioral response to 5-HT_2_ and NMDA receptor antagonists in MAOA KO mice may be sexually dimorphic. Third, although KET is a preferential 5-HT_2A_ antagonist, this drug blocks other 5-HT_2_ subtype receptors, including 5-HT_2B_ and 5-HT_2C_ (albeit with lower affinities). Therefore, our studies cannot exclude that these receptors may contribute to the serenic effects of KET or, possibly, to the overall behavioral effects of the combination between this drug and DIZ. At the same time, it is worth pointing out that most evidence suggests that 5-HT_2A_ (rather than 5-HT_2B_ or 5-HT_2C_) receptor antagonists block the effects of NMDA receptor antagonists (including NR2B-specific blockers). For example, Higgins et al. [64] showed that 5-HT_2A_ receptor antagonism attenuates behavioral outcomes of DIZ. Future studies will need to evaluate the combination of NMDA receptor blockers with selective 5-HT_2A_, 5-HT_2B_, and 5-HT_2C_ receptor antagonists. Finally, the analysis of the combined effects of KET and DIZ on motor behavior was only limited to the analysis of the activity in the context of aggression to rule out spurious findings. While a more comprehensive survey of the potential effects of the combination of KET and DIZ on movement is needed, other studies have already shown that the impairments of motor coordination and grip strength induced by this NMDA receptor antagonist were reversed by other 5-HT_2_ receptor antagonists [65].

These limitations notwithstanding, the results of this study point to the combination of non-subunit-selective NMDA and 5-HT_2_ receptor antagonists as a potential treatment option for aggression and antisocial behavior, at least in *MAOA*-deficient subjects.

## 4. Materials and Methods

Aggressive behavior is a major public health problem, and its therapy remains highly challenging. Here, we documented that the combination of DIZ (which shares the non-competitive NMDA antagonism with the FDA-approved drug ketamine) and KET may reduce aggression and antisocial behavior, with a better safety and tolerability profile than each monotherapy, in MAOA-deficient mice. Further studies will be needed to verify whether this pharmacological strategy may be effective against other neurobiological subtypes of pathological aggression.

## 5. Conclusions

### 5.1. Animal Husbandry

A total of 332 (*n* = 158 WT; *n* = 174 MAOA KO) experimentally naive 129S6 male adult (2–3 months old) mice, weighing 25–32 g, were used for this study. MAOA^A863T^ KO mice were generated as previously described [13]. Animals were housed in group cages (4–5 animal/cage) with *ad libitum* access to food and water. The room was maintained at the recommended temperature of ~18–23 °C and 40–60% humidity, and housed under a reversed light: dark cycle (lights off at 07.00 h and on at 19.00 h). Behavioral experiments occurred during the dark phase between 9 a.m. and 4 p.m. In all the experiments, male MAOA KO hemizygous mice were compared with their WT littermates. As the *MAOA* gene is an X-linked gene [66], male offspring of MAOA KO dams are either MAOA KO or WT. No heterozygous or homozygous male mice are available for this line of animals. Mice from at least three different litters were used to minimize potential litter effects. All experimental procedures were conducted in accordance with the National Institute of Health guidelines and approved by the Animal Use Committees of all Institutions.

### 5.2. Drugs

The following drugs were used: DIZ maleate (Sigma-Aldrich, St. Louis, MO, USA), Ro-256981 (Tocris Bioscience, Bristol, UK), KET (Sigma-Aldrich). DIZ and Ro-256981 were dissolved in 0.9% saline and administered intraperitoneally in an injection volume of 10 mL/kg. KET was dissolved in 10% β-Cyclodextrin, brought to volume with Ringer solution (1:5), and injected bilaterally into the mPFC in an injection volume of 0.1 μL (side).

### 5.3. Surgical and Drug Microinjection Procedures

Mice were anesthetized with a combination of chloral hydrate, magnesium sulfate, and pentobarbital sodium (Equithesin) and placed in a stereotaxic apparatus (Kopf Instruments, Tujunga, CA, USA). Under aseptic conditions, mice were shaved, and their scalp was retracted. A single midline burr hole was drilled. A double stainless steel guide cannula was inserted into the target area and secured with a drop of cyanoacrylate adhesive, and covered with dental cement. The following mPFC coordinates were selected based on the Paxinos and Franklin mouse brain atlas [67]: AP: +1.8 mm; ML: 0.0 mm; DV: −2.5 mm (from the skull surface). Following surgery, mice were housed in individual rectangular plastic cages, located side by side to prevent the influence of chronic stress due to isolation. Analgesic and antibiotic treatments were provided under the direction of the staff veterinarian. Mice were monitored daily until the sutures were removed and then once to twice weekly until completion of the study. Neither postoperative pain nor discomfort (decreased activity, decreased food, and water intake, weight loss, vocalizations, rough hair coat, hunched posture) were evidenced. However, two WT and three MAOA KO mice were excluded from the analyses due to their poor performance in the behavioral tests (startle reflex below the value of 100 AU in response to pulse stimuli). mPFC drug microinjections were carried out with stainless steel injectors, extending 1.0 mm below the tip of the guide cannulae. Injectors were attached to a 5 μL Hamilton syringe using PE-10 tubing, connected to a CMA microinjection pump. Following the infusion (0.05 μL/side), the injector remained in place for 1 min to allow the drug diffusion. On completion of testing, mice were sacrificed, and the locations of cannula tips were histologically verified by trained operators blind to behavioral results (Supplementary Figure S1). Three animals with errant locations of the targeted areas were excluded from the analysis.

### 5.4. Startle Reflex and PPI

Mice were tested as previously indicated [68]. Briefly, the apparatus (Med Associates, St Albans, VT, USA) consisted of four standard cages placed in sound-attenuated chambers with fan ventilation. Each cage consisted of a Plexiglas cylinder (diameter: 5 cm) mounted on a piezoelectric accelerometric platform connected to an analog–digital converter. Two separate speakers conveyed background noise and acoustic bursts, each properly placed to produce a variation of sound within 1 dB across the startle cage. Both speakers and startle cages were connected to a central PC, which detected and analyzed all chamber variables with specific software. Before each testing session, acoustic stimuli and mechanical responses were calibrated via specific devices supplied by Med Associates. The testing session featured a background noise of 70 dB and consisted of an acclimatization period of 5 min, followed by three consecutive sequences of trials (blocks). Unlike the first and the third block, during which mice were presented with only five pulse-alone trials of 115 dB, the second block consisted of a pseudorandom sequence of 50 trials, including 12 pulse-alone trials, 30 trials of pulse preceded by 74, 78, or 82 dB pre-pulses (10 for each level of prepulse loudness), and eight no-stimulus trials, where only the background noise was delivered. Inter-trial intervals were selected randomly between 10 and 15 s. %PPI was calculated using the following formula: 100 – ((mean startle amplitude for prepulse pulse trials/mean startle amplitude for pulse alone trials) × 100). The five pulse-alone trials in the first and third blocks were excluded from the calculation. PPI values related to different prepulse levels were collapsed, given that no interactions were found between prepulse levels and pharmacological treatments throughout the study. Throughout the PPI sessions, no-stimulus trials data were found negligible compared to other startle values; therefore, they will not be presented here. 

### 5.5. Resident–Intruder Aggression

Resident–intruder aggression was assessed as previously described [69]. Briefly, male mice were isolated in their home cages for fourteen days to establish territorial behavior. An unfamiliar age- and weight-matched male conspecific was placed in the home cage, and animals were allowed to interact freely for 5 min. Behavioral measures included the latency to the first attack, the total number of attacks, and the overall duration of fighting episodes. An attack was defined as a burst of bites, sideways threats, and rough grooming initiated by the resident. Intruders were only used once to avoid potential stress carryover effects. Overall locomotor activity was also assessed as the number of crossings of a 4 × 3 rectangular grid superimposed on the video image of each cage.

### 5.6. Statistical Methods

Data were expressed as mean ± SEM. Normality and homoscedasticity of data distribution were verified using the Kolmogorov–Smirnov and Bartlett’s tests. Potential outliers were confirmed by the Grubbs’ test. Parametric analyses were performed by two- or three-way ANOVA, as appropriate, followed by Tukey’s test (with Spjøtvoll–Stoline correction when necessary) for post hoc comparisons. The significance threshold was set at *p* = 0.05. 

## Data Availability

Data is contained within the article and Supplementary Materials.

## Supplementary Materials

The following supporting information can be downloaded at: https://www.mdpi.com/article/10.3390/ph15020213/s1: Figure S1: Histological verification of cannula placements in the mPFC. For further details see Material and Methods section.
