# Supplementary material for: Combined Antagonism of 5-HT2 and NMDA Receptors Reduces the Aggression of Monoamine Oxidase a Knockout Mice
Source: Pharmaceuticals (Basel). 2022 Feb 10;15(2):213. doi: 10.3390/ph15020213 (PMC8875523; doi:10.3390/ph15020213)
Supplement: Supplementary file 1 [file pharmaceuticals-15-00213-s001.zip › pharmaceuticals-1575999-supplementary.pdf]

Article

Combined Antagonism of 5-HT$_2$ and NMDA Receptors Reduces the Aggression of Monoamine Oxidase A Knockout Mice

Roberto Frau [1,2*], Alessandra Pardu [1], Sean C. Godar [3,4] Valentina Bini [1], and Marco Bortolato [4*]

[1] Department of Biomedical Sciences, Division of Neuroscience and Clinical Pharmacology, University of Cagliari, Monserrato (CA), Italy.
[2] *Guy Everett Laboratory*, Department of Biomedical Sciences, Division of Neuroscience and Clinical Pharmacology, University of Cagliari, Monserrato (CA), Italy.
[3] Dept. of Pharmacology and Pharmaceutical Sciences, University of Southern California, Los Angeles, CA, USA.
[4] Department of Pharmacology and Toxicology, College of Pharmacy, University of Utah, Salt Lake City, UT, USA.
* Correspondence: roberto.frau@unica.it; Marco.Bortolato@utah.edu

Supplementary material

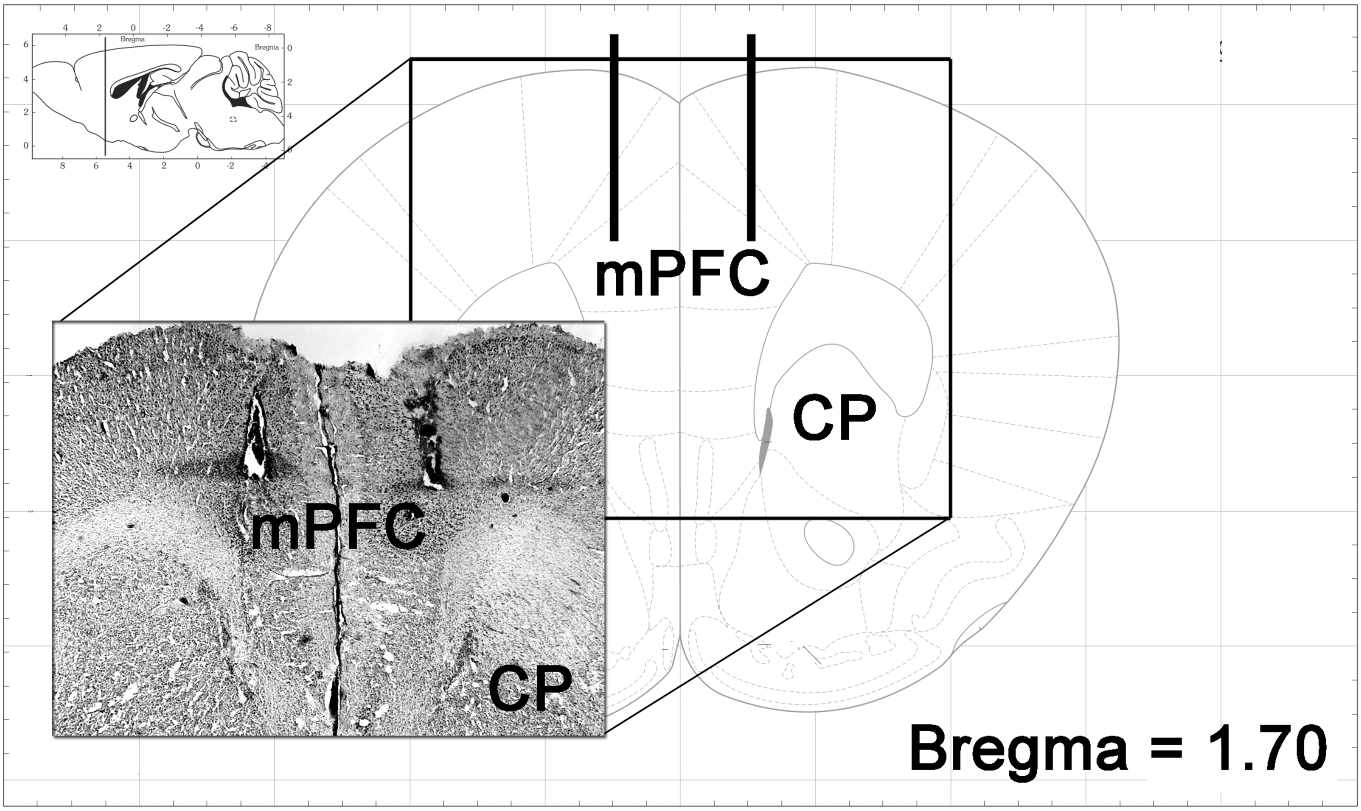

Figure S1. Histological verification of cannula placements in the mPFC.
